# Recipe for the Bite of a Mad Dog

**Published:** 1805-04-01

**Authors:** Thomas Hope

**Affiliations:** Chatham


### *To the Editors of the Medical and Physical Journal.*

GENTLEMEN,

IT is a source of satisfaction to observe, that whilst a number of your Correspondents are tossed on the stormy ocean of controversy, there are others pursuing the pleasurable course of calm investigation; and through the medium of your valuable publication, communicating useful and salutary information; among which, that of Dr. Bardsley requires particular attention; his narration of, of, and reflections on, the melanchly catastrophe related in No. 72, can but engage the feelings of every man of humanity, and excite the rising wish, that some remedy may be discovered that will remove so dreadful a disorder as that of rabies canina.

As the Doctor, in p. 163, expresses a hope that there is yet a remedy for this disorder, and seems disposed to try its effects, should it ever fall again to his lot to witness another such case; and that that remedy he judges to be arsenic: I have, to encourage the practice, sent you the following recipe; which I have for some time had in my possession. The following is a literal transcription, and is entitled,

*A Receipt for the Bite of a Mad Dog.*

Take the leaves of rue, pick them from the stalk, bruise them; six ounces of garlick, picked from the stalk, and bruise them likewise; Venice treacle, or mithridate, and the scrapings of pewter, of each four ounces.

Boil all the above ingredients over a slow fire, in two quarts of strong ale, till one pint be consumed; then keep it in a bottle, close stopt up, and give thereof to a man, or woman, (warm it first) nine spoons full, seven mornings fasting together.

To a dog six spoons full.

This, the author believes, will not, by God's blessing, fail, if it be given within nine days after the biting of a dog.—Apply some of the ingredients from which the liquor was strained to the bitten place.

This recipe was taken out of Colthorpe Church, in Lincolnshire, in 1766, the whole town being bitten by a mad dog; all that took this medicine did well, the rest that did not take it, died. Thus far the copy.

The quantity of rue is not mentioned, nor do I suppose it a matter of importance, the chief ingredient is most probably the pewter, and that from the quantity of arsenic which enters its composition. How far the character given of it may be depended on, cannot, perhaps, at this distance of time, be ascertained. Yet, I hope that neither Dr. B. nor yourselves, will think the communication altogether useless.

I am, &c.

